# High-Power Quantum Cascade Lasers Emitting at 8 μm: Technology and Analysis

**DOI:** 10.3390/nano12223971

**Published:** 2022-11-11

**Authors:** Evgeniia Cherotchenko, Vladislav Dudelev, Dmitry Mikhailov, Grigorii Savchenko, Dmitriy Chistyakov, Sergey Losev, Andrey Babichev, Andrey Gladyshev, Innokentiy Novikov, Andrey Lutetskiy, Dmitry Veselov, Sergey Slipchenko, Dmitry Denisov, Andrey Andreev, Irina Yarotskaya, Konstantin Podgaetskiy, Maksim Ladugin, Aleksandr Marmalyuk, Nikita Pikhtin, Leonid Karachinsky, Vladimir Kuchinskii, Anton Egorov, Grigorii Sokolovskii

**Affiliations:** 1 Ioffe Institute, 194021 St. Petersburg, Russia; 2 Connector Optics LLC, 194292 St. Petersburg, Russia; 3 Institute of Advanced Data Transfer Systems, ITMO University, 197101 St. Petersburg, Russia; 4 JSC MF Stelmakh Polyus Research Institute, 117342 Moscow, Russia

**Keywords:** high-power QCL, InP-contact layer, mid-infrared

## Abstract

In this work, we demonstrate the features of a two-stage epitaxial growth technique and show the results of power and efficiency measurements for three different designs of quantum cascade lasers with a record-high peak power in the 8 μm spectral region. The time-resolved QCL spectral study proves that InP-based upper cladding paired with an InP contact layer provides better heat dissipation and allows one to reach better power characteristics in comparison with InGaAs-based contact, even with short pulse pumping.

## 1. Introduction

First proposed in 1971 [1], quantum cascade lasers are devices that have become the leading platform for applications in the mid-infrared range, such as gas analysis, lidar systems, and many others. High-power QCLs are highly desired for infrared countermeasure technologies and optical communication developments, such as chaotic light sources [2], spectroscopy, sensing, and many others, so novel designs are underway [3] to increase the output power and efficiency characteristics of QCLs. The recent progress in the development of mid-infrared QCLs discussed in [4] shows that in the CW mode, the highest power values are obtained in the wavelength range close to 5 micrometers, while at higher wavelengths, the result is less impressive, as it is an order of magnitude lower. For the pulsed pumping regime, a similar picture can be observed [5]. At the same time, it is the >8 micrometer range that is of the main interest for wireless optical communication technologies due to the atmospheric transparency and low Rayleigh scattering in this wavelength range [6]. Thus, the development of novel QCL designs that improve the main important characteristics, such as power and efficiency, is highly desired. In this work, we discuss the two-stage MBE-MOCVD process, which allows the formation of an active region with upper cladding and contact layers in separate processes. The design with an InP-based contact layer demonstrated a record-high peak power for 8 μm emitting QCLs. We claim that this result is due to the better heat dissipation even with short pulsed pumping, and we prove this with measurements of the active region’s heating dynamics.

## 2. Theoretical Estimation and Methods

High internal optical losses, which are responsible for the characteristics of low efficiency and power, are associated with resistive heating and free carrier absorption (FCA) processes and should be taken into account when designing not only the active region, but also the claddings and contact layers of QCLs. The right design of the latter two components can significantly improve the laser’s performance. It is known that FCA strongly depends on the optical confinement factor Γ and the density of impurity electrons in the upper cladding layer:

(1) $$\alpha_{fc}=\Gamma \sigma N,$$

where σ is the absorption cross-section for the given wavelength and *N* is the concentration of the impurity electrons.

**Figure 1:**
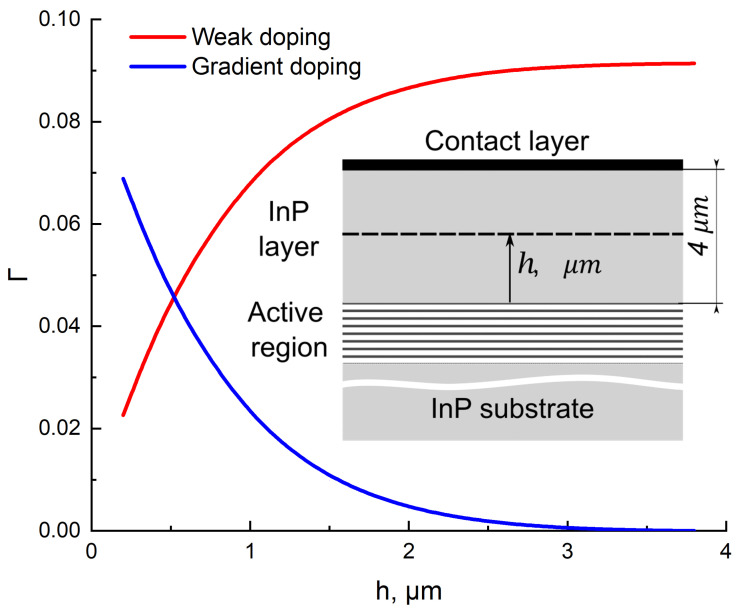
The optical confinement factor Γ in the InP waveguide layers as a function of the distance from the active region. The red curve indicates the weakly doped InP layer, and the blue curve corresponds to a gradient doping varying from 1 × 10${}^{16}$ to 1 × 10${}^{19}$ cm${}^{-3}$. The inset schematically illustrates the structure: h indicates the distance from the active region and, simultaneously, the thickness of the weakly doped region of the InP layer.

To ensure the flow of current in the system, we consider the inhomogeneous doping of the upper InP cladding with an overall thickness of 4 μm. Over the first *h* μm from the active region, the impurity concentration is 10${}^{16}$ cm${}^{-3}$, which is a rather low doping; then, it gradually increases to 10${}^{18}$ cm${}^{-3}$. We denote by $\Gamma_{1}$ and $\Gamma_{2}$ the optical confinements of these two regions. By varying the thickness ratio of the two parts, we calculate $\Gamma_{1,2}$ and estimate the thickness of the upper cladding to reduce the FCA losses. To guarantee this, the Γ-factor of the weakly doped layer must be of an order of magnitude higher than the highly doped one. In our estimations, to satisfy this condition, the ratio of the two thicknesses is ≥2.5/1.5. Figure 1 illustrates this idea. The total InP thickness is 4 μm, the sum of Γ-factors for both parts (highly and weakly doped) is constant, and one can clearly see that in the region where h≥2.5, the Γ-factor of the highly doped layer is significantly reduced. This result is independent of the type of upper contact due to the significant thickness of the cladding layer.

The study of the resistive heating problem is more complicated from both the theoretical and experimental points of view. Most experimental techniques are based on surface studies, which allow the measurement of the temperature only on the laser facet [7,8]. Theoretical estimations that include temperature consideration usually examine only the active region and do not include claddings and contacts. To take the latter into account, one should produce quite complicated numerical modeling, as was done in [9]. We used qualitative conclusions from the latter for further investigations.

Following the estimations, we developed three different QCL designs with different parameters of the upper cladding/near-contact layer pairs, leaving the active region the same in all structures. To make the fabrication more cost- and time-efficient, as discussed in [10], the growth of the samples was performed in two separate steps. Initially, we used the phosphorus-free MBE technique to grow the active region in a process similar to that reported in [11,12]. The last MBE-grown layer was 50 nm InGaAs, which was designed to cover the active region with 50 quantum cascades. After that, the process was interrupted, and a ∼4 μm thick upper cladding with a highly doped contact layer was grown through MOCVD. Table 1 shows detailed descriptions of these three designs. The detailed investigation proved that the heterostructures obtained were of the same quality as the ones in [12], which were grown in a single-stage MBE process. All 3 heterostructures were subjected to a post-growth fabrication of QCL chips with 40 µm stripes and 3 mm cavity lengths. All samples were tested under 150 ns pulsed pumping with a 12 kHz repetition rate.

## 3. Experimental Results

**Figure 2:**
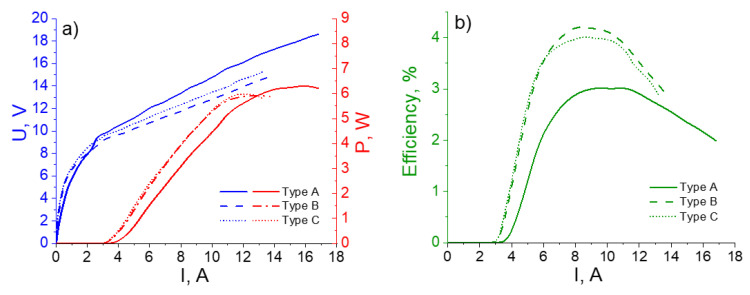
The L(I) and V(I) (**a**) and efficiency (**b**) curves measured for the lasers based on the three types of heterostructures. The details on the latter are shown in Table 1. The QCLs of Type C and Type B demonstrated lower threshold characteristics and better efficiencies. However the peak power was the highest for the Type A QCL due to the better thermoconductivity of the upper cladding.

Figure 2 shows the typical light–current, voltage–current, and efficiency characteristics of the samples of Types A, B, and C. The peak optical power of ≈6 W (0.9 μJ pulse energy) was observed for the QCLs of Types A–C. However, the highest power was demonstrated with the Type A structure featuring the InP contact layer. In Figure 2b, one can see that better efficiency and lower threshold currents appeared for the heterostructures of Types B and C. The highest efficiency was reached in the structure that was designed with the gradient doping applied over the 2 microns of the upper cladding. The latter can be explained by the better electrical conductivity provided by the InGaAs contact layer. At the same time, the peak power strongly depended on the QCL temperature. The thermal conductivity of the InP layer was ≈15 times higher than that in the InGaAs materials [13]. Thus, the Type A QCL with the InP contact layer was heated more slowly than those based on the Type B and Type C heterostructures. To show this, we studied the time-resolved QCL spectra to estimate the influence of the contact layer’s thermal conductivity on the heating of the active region of the QCL. The main feature of this experimental technique was the ability to find the change in temperature inside the active region.

**Figure 3:**
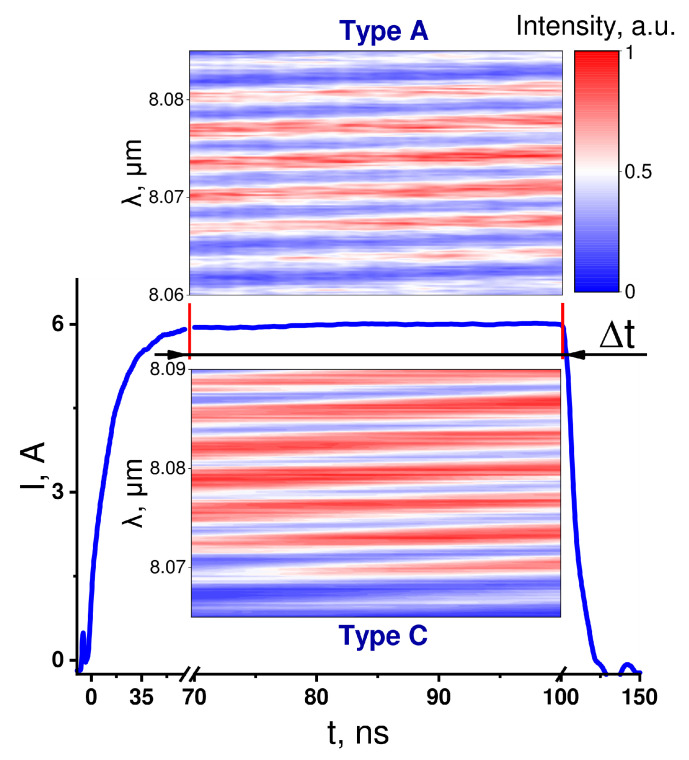
The measurements of the heating rate in the QCLs based on two different heterostructures: Type A with the InP-based upper cladding and contact layer (the upper colored panel) and Type C with the InP-based upper cladding and InGaAs-based contact layer (the lower colored panel). The colored panels are heatmaps that show the intensity of the Fabry–Perot modes as a function of time and wavelength. The blue curve in the middle shows the pump pulse. Δt is the time range during which the heating rate was measured. It corresponds to a nearly constant pumping value. The maximum pumping current reached 6 A.

Figure 3 shows the lasing intensity as a function of time and wavelength. The method was based on measuring the chirp of the Fabry–Perot modes in a QCL resonator, which occurred due to the heating of the QCL active region. The laser emission was collected at the monochromator slit and recorded via a high-speed mid-IR photodetector and oscilloscope, with both featuring a 1 GHz bandwidth. The combination of the waveforms recorded for each spectral step made it possible to build time-resolved QCL spectra, such as those shown in the colored panels of Figure 3. These spectra were used to determine the chirp values. Then, the heating rate was calculated by using the measured red shift of the Fabry–Perot modes, which was associated with an increase in the refractive index when the active region was heated. The heating rate was estimated by using the simple expression [14]:

(2) $$\frac{dT}{dt}=\frac{\partial n}{\partial t}{\left(\frac{\partial n}{\partial T}\right)}^{-1}=\frac{n}{\lambda_{m}}\frac{\partial \lambda_{m}}{\partial t}{\left(\frac{\partial n}{\partial T}\right)}^{-1}.$$

Here, *n* is the refractive index of the active region, and $\lambda_{m}$ is the wavelength of the peak intensity of the particular Fabry–Perot mode. The measurements were performed in the region in which the amplitude of the pumping pulse with a total duration of 150 ns could be considered as constant. Our measurements indicate that the heating rate of the structure with the InP-based upper cladding and contact layer was nearly 20% lower than that in the structures with an InGaAs contact layer. Notably, the difference in heating rates tended to increase with higher pumping.

*Efficiency and waveguide losses*:

The better efficiency characteristics observed in the structures of Types B and C triggered the further investigation of these designs. The main difference was the non-uniform gradient doping of the upper cladding layer adjacent to the contact in the Type A structure. The waveguide losses could be estimated from the well-known expression:

(3) $$\frac{1}{\eta_{d}}=\frac{2}{\tilde{\eta }\eta_{i}}\left(1-\frac{\alpha_{w}L}{lnR}\right),$$

where *R* is a reflectance coefficient, *L* is the cavity length, $\tilde{\eta }$ is the pumping efficiency, $\eta_{i}$ is the internal quantum efficiency, $\alpha_{w}$ is the waveguide losses, and $\eta_{d}$ is the differential quantum efficiency (DQE). The intercept $\frac{2}{\tilde{\eta }\eta_{i}}$ could be found from the extrapolation of the experimental data. The gradient of the inverse slope efficiency is given by

(4) $$\theta =\frac{2}{\tilde{\eta }\eta_{i}}\frac{\alpha_{w}}{ln\frac{1}{R}}.$$

Measuring $\eta_{d}$ and calculating θ, we found the value of the waveguide losses $\alpha_{w}$.

**Figure 4:**
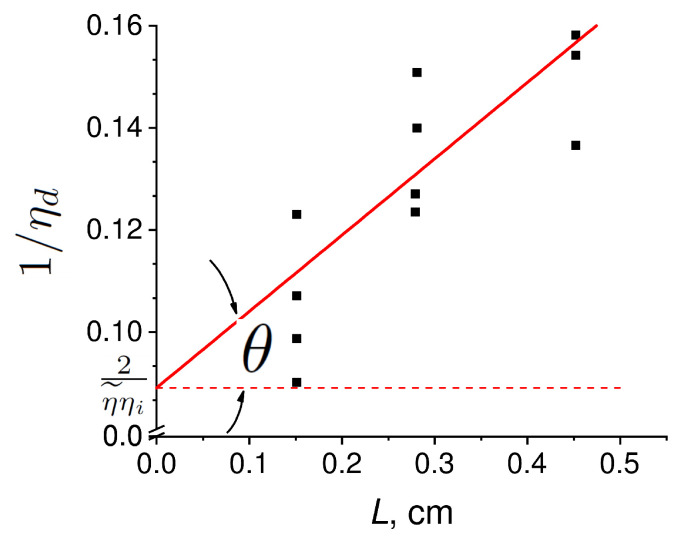
The dependence of the inverse slope efficiency on the cavity length. The black squares show experimental data. The red line is the data line approximation.

The dependence of the inverse slope efficiency on the cavity length is shown in Figure 4. The red line shows the linear fit of the experimental values. θ was found to be 0.15 ± 0.04, with $2/\tilde{\eta }\eta_{i}$ being its value for a laser with zero cavity length. This intercept is found to be 0.09 ± 0.01. The estimated value of the waveguide losses was 2.1 ± 0.7 cm${}^{-1}$, which was a relatively good result [15].

**Figure 5:**
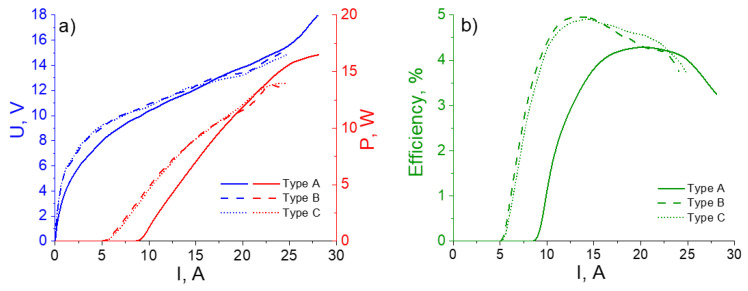
The L(I) and V(I) (**a**) and efficiency (**b**) curves measured for the 60 μm QCL stripes.

*High-power experiments*: Based on the L(I) measurements of the QCL stripes of the 40 μm width, the structures were used to form 60 μm stripes. All samples were tested again under 150 ns pulsed pumping with a 12 kHz repetition rate. In Figure 5, we show the new L(I), V(I), and efficiency characteristics for the QCLs fabricated from the structures of Types A–C with a 60 μm stripe. One can see the peak optical power of over 16.5 W (>8 W/facet, 2.5 μJ pulse energy) reached in the QCLs of Type A with an InP contact layer, which is the record power in this wavelength range to the best of our knowledge [5]. At the same time, the tendency of the efficiency curves was conserved: The structures with the InGaAs contact layer still showed higher values.

## 4. Discussion

In this work, we discussed several designs that showed the competition between increased efficiency and high power for a pulse-pumped QCL emitting in the 8 μm range. On the one hand, it is evident that better heat removal leads to a better QCL performance, even with short pulse pumping, which may seem counterintuitive with respect to the experimental behavior of laser diodes. There have been a few attempts to design efficient heat-sinking configurations [16,17,18]. These technologies are mainly based on thick electroplated Au, a buried heterostructure, and epilayer-down mounting. In [9], the authors discussed epilayer-up mounting with different means of post-growth heat dissipation. This work is extremely important for reaching the CW-mode in a QCL device. At the same time, all of these configurations are technologically complicated and not always economically justified when one needs QCLs to work in a pulsed regime. In the case without InP regrowth, the only means of heat propagation was through the upper cladding, with possible heat removal by the heat sink with epilayer-down mounting. At the same time, as mentioned at the beginning, the internal losses do not depend on the type of upper contact, while the efficiency is heavily dependent on the quality of the electrical contact. Thus, the replacement of a conventional InGaAs contact featuring high electroconductivity with highly doped InP may lead to improved power and efficiency characteristics. In our experiments, the doping was lower than that needed for good electrical contact; as a result, we observed, higher threshold currents together with a high peak power. Future work will include an increase in InP contact doping to improve the threshold characteristics for structures of Type A. Apart from that, from our first point of view, looking at the Figure 5, it seems that gradient doping has nothing to do with efficiency. In any case, we would like to point out that in structures with narrower stripes, where the currents are lower and the thermal effects are not so pronounced, the efficiency of structure B with gradient doping is higher than in the uniformly doped structure C, although the contact layer is the same in both cases. This result can be seen in Figure 2, and this is exactly why we think this design can be promising for more efficient QCL performance and needs further exploration.

## 5. Conclusions

The two-step growth technique developed here allowed the design and fabrication of different QCL heterostructure designs with remarkable output power in the pulsed mode. The quality of the abricated heterostructures was similar to that of structures produced with the one-stage MBE technique. As a result, we demonstrated a design that features an increased peak power of QCLs emitting at 8 μm due to the better thermoconductivity. The maximum peak power reached ≥16 W, which is the record-high value for this wavelength range, to the best of our knowledge. As a future perspective, we plan to increase the InP contact layer doping to improve the efficiency characteristics of the QCL.

## Figures and Tables

**Table 1:** Upper cladding/contact layer designs. The QC configuration used for the structures of Types A–C comprised a superlattice of alternating **In${}_{0.53}$Ga${}_{0.47}$As**/Al${}_{0.48}$In${}_{0.52}$As layers that were lattice-matched with the InP substrate [11]. The thicknesses of the sequential layers were **2.4**/2.4/**2.6**/2.1/**2.6**/1.8/**2.7**/1.6/**2.9**/1.7/**3.1**/2.5/**4.4**/ 1.2/**5.2**/1.2/**5.3**/1.0/**1.7**/4.3 nm.

	Composition	Thickness, nm	Doping, cm${}^{-3}$
**Type A**			
Contact Layer	InP	20	1 × 10${}^{18}$
Upper Cladding	InP	4000	1 × 10${}^{17}$
**Type B**			
Contact Layer	In${}_{0.53}$Ga${}_{0.47}$As	20	1 × 10${}^{19}$
Upper Cladding (2)	InP	2000	gradient 1 × 10${}^{16}$–1 × 10${}^{18}$
Upper Cladding (1)	InP	2000	1 × 10${}^{16}$
**Type C**			
Contact Layer	In${}_{0.53}$Ga${}_{0.47}$As	20	1 × 10${}^{19}$
Upper Cladding	InP	4000	1 × 10${}^{17}$

## Data Availability

Not applicable.

## References

[1] Kazarinov R.F., Suris R.A. (1971). Possibility of amplification of electromagnetic waves in a semiconductor with superlattice. Sov. Phys. Semicond..

[2] Jumpertz L., Schires K., Carras M., Sciamanna M., Grillot F. (2016). Chaotic light at mid-infrared wavelength. Light Sci. Appl..

[3] Botez D., Kirch J.D., Boyle C., Oresick K.M., Sigler C., Kim H., Knipfer B.B., Ryu J.H., Lindberg D., Earles T. (2018). High-efficiency, high-power mid-infrared quantum cascade lasers. Opt. Mater. Express.

[4] Figueiredo P., Suttinger M., Go R., Tsvid E., Patel C.K.N., Lyakh A. (2017). Progress in high-power continuous-wave quantum cascade lasers. Appl. Opt..

[5] Zhou W., Lu Q.Y., Wu D.H., Slivken S., Razeghi M. (2019). High-power, continuous-wave, phase-locked quantum cascade laser arrays emitting at 8 μm. Opt. Express.

[6] Lavery M.P.J., Peuntinger C., Günthner K., Banzer P., Elser D., Boyd R.W., Padgett M.J., Marquardt C., Leuchs G. (2017). Free-space propagation of high-dimensional structured optical fields in an urban environment. Sci. Adv..

[7] Pierścińska D. (2017). Thermoreflectance spectroscopy–Analysis of thermal processes in semiconductor lasers. J. Phys. D Appl. Phys..

[8] Wang S., Xu C., Duan F., Wen B., Rassel S.M.S., Tam M.C., Wasilewski Z., Wei L., Ban D. (2022). Thermal dynamic imaging of mid-infrared quantum cascade lasers with high temporal-spatial resolution. J. Appl. Phys..

[9] Lee H.K., Chung K.S., Yu J.S., Razeghi M. (2009). Thermal analysis of buried heterostructure quantum cascade lasers for long-wavelength infrared emission using 2D anisotropic heat-dissipation model. Phys. Status Solidi (A).

[10] Wang F., Slivken S., Wu D.H., Lu Q.Y., Razeghi M. (2020). Continuous wave quantum cascade lasers with 5.6 W output power at room temperature and 41% wall-plug efficiency in cryogenic operation. AIP Adv..

[11] Babichev A.V., Gladyshev A.G., Filimonov A.V., Nevedomskii V.N., Kurochkin A.S., Kolodeznyi E.S., Sokolovskii G.S., Bugrov V.E., Karachinsky L.Y., Novikov I.I. (2017). Heterostructures for quantum-cascade lasers of the wavelength range of 7–8 μm. Tech. Phys. Lett..

[12] Dudelev V.V., Losev S.N., Mylnikov V.Y., Babichev A.V., Kognovitskaya E.A., Slipchenko S.O., Lutetskii A.V., Pikhtin N.A., Gladyshev A.G., Karachinskii L.Y. (2018). Dual-Frequency Generation in Quantum Cascade Lasers of the 8 μm Spectral Range. Opt. Spectrosc..

[13] Jaffe G.R., Mei S., Boyle C., Kirch J.D., Savage D.E., Botez D., Mawst L.J., Knezevic I., Lagally M.G., Eriksson M.A. (2019). Measurements of the Thermal Resistivity of InAlAs, InGaAs, and InAlAs/InGaAs Superlattices. ACS Appl. Mater. Interfaces.

[14] Dudelev V.V., Mikhailov D.A., Babichev A.V., Mylnikov V.Y., Gladyshev A.G., Losev S.N., Novikov I.I., Lyutetskiy A.V., Slipchenko S.O., Pikhtin N.A. The Technique for QCLs Heating Dynamics Measurements. Proceedings of the 2020 International Conference Laser Optics (ICLO).

[15] Kirch J.D., Chang C.C., Boyle C., Mawst L.J., Lindberg D., Earles T., Botez D. (2015). Highly temperature insensitive, low threshold-current density (*λ* = 8.7–8.8 μm) quantum cascade lasers. Appl. Phys. Lett..

[16] Yu J.S., Slivken S., Evans A., David J., Razeghi M. (2003). Very high average power at room temperature from *λ*≈5.9 μm quantum-cascade lasers. Appl. Phys. Lett..

[17] Beck M., Hofstetter D., Aellen T., Faist J., Oesterle U., Ilegems M., Gini E., Melchior H. (2002). Continuous Wave Operation of a Mid-Infrared Semiconductor Laser at Room Temperature. Science.

[18] Gmachl C., Sergent A., Tredicucci A., Capasso F., Hutchinson A., Sivco D., Baillargeon J., Chu S., Cho A. (1999). Improved CW operation of quantum cascade lasers with epitaxial-side heat-sinking. IEEE Photonics Technol. Lett..

